# Effects of Climate Change on Health and Health Systems: A Systematic Review of Preparedness, Resilience, and Challenges

**DOI:** 10.3390/ijerph22020232

**Published:** 2025-02-06

**Authors:** Vasileios Gkouliaveras, Stavros Kalogiannidis, Dimitrios Kalfas, Stamatis Kontsas

**Affiliations:** 1 Department of Business Administration, School of Economic Sciences, University of Western Macedonia, 51100 Grevena, Greece; dba00040@uowm.gr (V.G.); skontsas@uowm.gr (S.K.); 2 Department of Agriculture, School of Agricultural Sciences, University of Western Macedonia, 53100 Florina, Greece

**Keywords:** climate change, health systems, sustainable health system, health care adaptation strategies, resilience challenges, climate change mitigation policies

## Abstract

Climate change has a significant impact on the population’s health and negatively affects the functioning of healthcare systems. Health systems must be operationally prepared to handle the challenges posed by environmental change. Resilience is required to adapt quickly to critical environmental conditions and reduce carbon emissions. In this systematic review strategies, for health system preparedness and resilience are examined to address the impacts of climate change, and the barriers and challenges faced when implementing them. To identify studies, the Scopus, PubMed and Google Scholar databases were searched three times (from April to October 2024, 21 April, 15 June, and 9 September) for the years 2018 to 2024, using the PRISMA (Preferred Reporting Items for Systematic Reviews and Meta-Analyses) methodology. Specifically, the search identified 471 articles, of which the specified inclusion and exclusion criteria (secondary studies with inclusion criteria, being in English, etc.) were met by sixteen (16) studies. According to the findings of the studies reviewed, adaptation strategies focus on structural changes, the development of training programs, the development of surveillance systems, and appropriate operational plans. The leader’s ability to motivate employees to achieve defined goals, continuous evaluation of goals and interventions, and learning from previous disasters play an important role in their implementation. Similarly, key policies and strategies for mitigation include the adoption of sustainable practices, such as recycling and cultural change. However, lack of resources (human, material, financial) and increased demand for health services make it difficult to implement adaptation and mitigation strategies. The findings of the review are mainly theoretical in nature and are confirmed by other studies. It is suggested that further research on resilience and preparedness of health systems should be pursued, leading to their sustainability and the formulation of appropriate policies.

## 1. Introduction

### 1.1. Climate Change: Identifying Causes, Wider Impacts

According to the Intergovernmental Panel on Climate Change (IPCC) and the United Nations, climate change is one of the most significant global challenges of the 21st century [1] and a major obstacle to achieving the UN Sustainable Development Goals [2]. It is defined by the United Nations Framework Convention on Climate Change (UNFCCC) as “a change in climate that is directly or indirectly attributable to human activity that alters the composition of the global atmosphere and that is in addition to the natural climate variability observed over comparable time periods” [3].

As asserted by the argument, climate change is directly caused by human activity, including deforestation, urbanization, high consumption/waste, transportation, and burning of fossil fuels and biomass. Consequently, the amount of CO_2_ accumulating in the atmosphere is increasing. The global transportation sector alone experienced a 45% increase in CO_2_ emissions from 1990 to 2007, while a further 40% increase is expected by 2030 [4]. Furthermore, climate change has a significant effect on the health sector, as it contributes around 4.4% of global greenhouse gas emissions [5,6,7]. In addition, the health sector has a significant impact on climate change, contributing approximately 4.4% of global greenhouse gas emissions [5,6,7]. These emissions come from the operational activities of health facilities (boilers, electricity generation), as well as from medicines and waste disposal [8].

The consequences of the above-mentioned situation are sea level rise [9] and warming of the earth’s surface, air, and water. The increase in climate variability [10] is a result of precipitation, storms, droughts [11], floods, hurricanes, and forest fires that are intense [12,13].

The impact of increasing temperature and extreme weather on economic activity is significant [14], negatively affecting labor productivity, slowing down investment, harming human health [15], and impacting the well-being and functioning of ecosystems [16]. It is worth noting that climate change poses the greatest risk to ecosystems worldwide [17]. In particular, the impact on marine biodiversity and ecosystem function, which, in combination with overfishing and coastal development, causes changes in physiology, abundance, genetic structure, and interactions with other species, has been well documented [18]. In addition, severe impacts on plants, animals, and overall biodiversity are predicted for the African continent [17]. These situations negatively affect agriculture, social livelihoods, and migration, and exacerbate social conflict [19]. Consequently, the benefits of ecosystems for society are altered [20].

More specifically, in South Africa, extreme levels of poverty make the population vulnerable to the effects of climate change. High temperatures and reduced rainfall will worsen crop yields, exposing 25% of the population to food insecurity, poor air quality, and disease (malaria). It is worth noting that in 2015, 4% of deaths were attributed to air pollution [21]. A similar picture in crops with an increase in malnutrition is observed in savanna zones such as Ghana [2] and Kiribati [22]. In Ethiopia, the 2015 drought led to an 80% crop loss, leaving 8 million people food insecure (40,000 children and 700,000 pregnant and lactating women at risk of malnutrition) [23].

In Pakistan, drought, severe storms, and floods have an impact on water quality, associated with an increase in communicable diseases such as cholera, typhoid, dengue, hepatitis, and malaria [24]. A similar picture prevails in India, with an increase in both communicable and non-communicable diseases [25], and in Nepal, with an increase in morbidity and mortality, especially among poor women, children, and the elderly [26]. In addition, the Caribbean is particularly vulnerable to extreme climatic events (hurricanes), which can cause human mobility (internal displacement and international migration) [27].

In contrast, in developed countries such as the UK, climate change will have an impact by increasing food prices. It is worth noting that in 2003, after the European heatwave, the fruit harvest decreased by 25%, so the increased prices will lead people to consume low-nutrition foods with health consequences (cardiovascular disease, obesity) [28]. In Canada, drought will affect mental health, respiratory disease, and water security. It will also increase injuries, infectious diseases, and mental illness [29]. Further increase in temperature and drought are predicted for the Mediterranean countries [30]. In Italy, the effects of climate change, and in particular heatwaves, have taken a toll on mental health, with an increase in morbidity (psychiatric hospitalizations) and suicides [31]. West Nile fever in southeastern Europe has also been associated with very high temperatures [32]. Therefore, rising temperatures lead to a reduction in freshwater resources, desertification, land use change, biodiversity loss, and environmental degradation [19]. The above-mentioned situations act as multipliers for the threat of food insecurity [22] as well as for communicable and non-communicable diseases [19]. The epidemiological cycle of zoonotic infectious diseases includes the host–pathogen and usually a potential intermediate vector. The effect of changing environmental conditions is expected to lead to the emergence and re-emergence of diseases in both developing and Western countries, putting the lives of millions of people at risk [33]. In recent years, health economists have focused their attention on studies related to the effects of climate change. Specifically, their work addresses the impacts of extreme weather events, extreme temperatures, atmospheric air pollution, and vector-borne diseases [34].

While the causes and impacts of climate change are well documented, its effects are often disproportionately distributed across regions and populations [21,35]. Vulnerable groups, including those in low-income countries, coastal communities, and indigenous populations, bear the brunt of these impacts due to limited resources, infrastructure, and adaptive capacity [36,37]. For instance, small island developing states (SIDSs) face existential threats from rising sea levels, while sub-Saharan Africa experiences intensified droughts and crop failures, exacerbating food insecurity and malnutrition [38]. Additionally, urban areas in developing countries often witness increased air pollution and heat stress, due to inadequate urban planning and reliance on fossil fuels [39,40].

Health systems play an important role in managing health risks and ensuring the well-being of citizens [41]. They are also affected by the effects of climate change. In the UK, in particular, heatwaves are associated with problems with medical equipment and the storage of medicines. They increase patient and staff suffering, demand for health services, and mortality in vulnerable populations such as the elderly. Similarly, problems caused by cold, snow, ice, and flooding affect patient transport services, making it harder for patients to access health facilities and for ambulances to respond quickly. The demand for such services has also increased due to injuries [42]. In Ethiopia’s health system, the challenges are different. In particular, there is a lack of access to health services for citizens, low availability of health facilities, and a shortage of human resources [23]. These disparities underscore the need for targeted and inclusive climate policies that address the unique vulnerabilities of these populations while fostering global cooperation to mitigate the shared risks of climate change [43,44].

Furthermore, the economic impacts of climate change extend beyond direct effects and create spillover effects across industries and sectors [45,46,47]. For example, extreme weather events disrupt global supply chains, increase insurance premiums, and require increased government spending on disaster relief and recovery [48,49]. The agricultural sector, which is highly dependent on stable weather patterns, suffers from reduced crop yields, leading to volatile food prices and increased poverty [50,51]. Similarly, infrastructure damage from storms and floods imposes significant costs on both public and private entities [52,53].

These cascading effects highlight the interconnectedness nature of climate, economy, and society, and underscore the urgency of adopting comprehensive and adaptive strategies to combat climate change and promote resilience across all levels of government and industry [54,55]. Based on the above, the first global legally binding agreement, related to action on adaptation and mitigation actions to climate change is the Paris Agreement [56]. Under this agenda, expectations have been created and raised around the world that institutions, governments, and businesses will be held accountable for advancing sustainable development [57].

### 1.2. Impacts of Climate Change on Health and Health Systems

Climate change poses an urgent and imminent global threat to human health [58], increasing morbidity and mortality, straining health services worldwide [59,60], and exacerbating existing social inequalities [61]. According to a baseline socio-economic scenario, climate change will have caused 250,000 deaths from malnutrition, heat stress, malaria, and diarrhea by 2030 and 33,000 child deaths from diarrheal diseases by 2050 [62].

These changing health needs will increase the demand for health services, costs [61], and migration [63]. The disruption of physical infrastructure and facilities, supply chains [64], and services contribute to the call for reducing greenhouse gas emissions from anthropogenic activities by 2030 and zero by 2050 [61].

It is essential to intensify and strengthen efforts to address the impacts of climate change [65]. Health service organizations must develop climate change-resistant health systems, identify vulnerabilities, and implement adaptation policies to achieve long-term sustainability [66]. Efforts to reduce the adverse effects of climate change [67] can be achieved through awareness raising, risk assessment, design, implementation, monitoring and evaluation of interventions [4], and mitigation policies [68] to reduce the global carbon footprint [66]. According to the Intergovernmental Panel on Climate Change, mitigation policies should focus on both the supply and the demand side (focusing on prevention and management of chronic diseases) [34].

The link between climate change and global health is further strengthened by the increasing prevalence of climate-sensitive diseases [69,70]. For instance, vector-borne diseases, such as malaria and dengue, are expanding into previously unaffected regions due to changing temperature and precipitation patterns. Similarly, respiratory illnesses, including asthma and chronic obstructive pulmonary disease (COPD), are exacerbated by worsening air quality and increased exposure to allergens like pollen [71,72]. Vulnerable populations, including children, the elderly, and those with pre-existing health conditions, are disproportionately affected, further straining already overburdened health systems [73]. These dynamics underscore the critical need for proactive health interventions and integrated climate-health policies [74,75].

In addition to the direct health impacts, climate change poses systemic challenges to healthcare delivery and infrastructure. Hospitals and clinics in disaster-prone areas are increasingly vulnerable to physical damage, power outages, and water shortages, compromising their ability to provide essential services during crises [76,77]. Moreover, the disruption of pharmaceutical supply chains, due to extreme weather events, can lead to shortages of critical medications, impacting the continuity of care for chronic diseases [78,79]. Addressing these challenges requires a paradigm shift toward climate-resilient health systems, characterized by sustainable resource management, enhanced disaster preparedness, and robust healthcare infrastructure, capable of withstanding and adapting to the pressures of a changing climate [80,81].

According to the World Health Organization, a climate-resilient health system is defined as “the ability, capability and capacity of the health system to predict, prevent, prepare, absorb, adapt and transform when exposed to shocks and stresses and deliver routine health services continuously during the crisis management” [82]. On this basis, healthcare systems need to prepare for crisis management during the planning, response, and recovery phases [58], taking into account the factors of their external environment (political, economic, legal, environmental, technological, and social) [11]. The present systematic review, which covers the last five years, adds significant benefits to the summary and understanding of mitigation and adaptation strategies, which makes it original. This is evidenced by its difference from other studies, specifically in terms of methodology [83,84] and its particular focus [1,58,84,85].

Therefore, the purpose of this systematic review is to summarize the knowledge in existing literature on how health systems are prepared and resilient to the impacts of climate change. It focuses on the strategies and practices, proposed to address the challenges of climate change and the barriers and challenges faced by healthcare systems. The following research questions were posed to achieve the above objective.

1. What strategies and practices are being implemented to strengthen the preparedness and resilience of health systems, according to the literature?
2. What are the main barriers and challenges to strengthening the preparedness, sustainability, and resilience of health systems, according to the existing literature?

## 2. Methods

A comprehensive and detailed search strategy plan has been created to reduce, biased errors by identifying, evaluating, and synthesizing all relevant studies on a particular topic [86]. The Preferred Reporting Items for Systematic Reviews and Meta-Analyses (PRISMA) 2020 guideline was implemented, to identify the methodologies used by the researchers and their findings to achieve the above [87].

### 2.1. Search Strategy

The literature search, as reflected in Table 1, was conducted using the most widely used academic databases, such as Scopus, PubMed, and Google Scholar, using keywords [(“Climate change” or “Environmental change”) and (“Health systems or health care”) and (“Preparedness or readiness”) and (“Sustainable or resilient”)]. These three databases were selected to minimize bias errors. The list included English-language studies published and peer-reviewed between 2018 and 2024. The language was chosen because most studies are conducted in English, significantly reducing bias error, and because other languages are not understood by the authors. The search process was conducted three times: on 21 April 2024, 15 June 2024, and 9 September 2024. The aim was to include as many studies as possible, including those that may not have been published yet.

### 2.2. Inclusion and Exclusion Criteria

The systematic review, as presented in Table 2, used specific inclusion and exclusion criteria, to identify relevant studies. These criteria were designed to ensure the review focused on high-quality, contemporary research that addressed the defined research questions. Key elements of the inclusion criteria included:

1. Secondary studies that have focused on the purpose of the study and the defined research questions
2. Studies that have used inclusion and exclusion criteria in their methodology
3. Published studies conducted in English
4. Studies published in peer-reviewed journals between 2018–2024, as this timeframe captures the most recent research on the topic
5. Studies whose consideration of their full content is relevant to the purpose of the study
6. Exclusion criteria for the systematic review include books and gray literature (theses, conference proceedings, reports, etc.)

### 2.3. Justification of Criteria

Concerning the justification of the criteria, there are the following six points:

1. Secondary studies were chosen because they can capture, combine, and summarize data from several primary studies, providing an overall picture of the topic.
2. Studies that have used inclusion and exclusion criteria in their methodology are more likely to lend greater validity and reliability to the study.
3. Only individual published studies in English were used because it is the most widely used language worldwide, both scientifically and communicatively.
4. The time frame 2018–2024 was chosen to reflect the most recent data from the literature on the topic.
5. Studies, whose general content is relevant to the topic, are those that lend validity and answer research questions.
6. The decision to exclude gray literature and books (theses, conference proceedings, reports) was taken because it is very difficult to both evaluate them and assess their reliability.

### 2.4. Selection Procedure

In the first phase, 471 studies were identified, as shown in Table 3, 301 in Google Scholar, 113 in PubMed, and 57 in Scopus.

**Figure 1:**
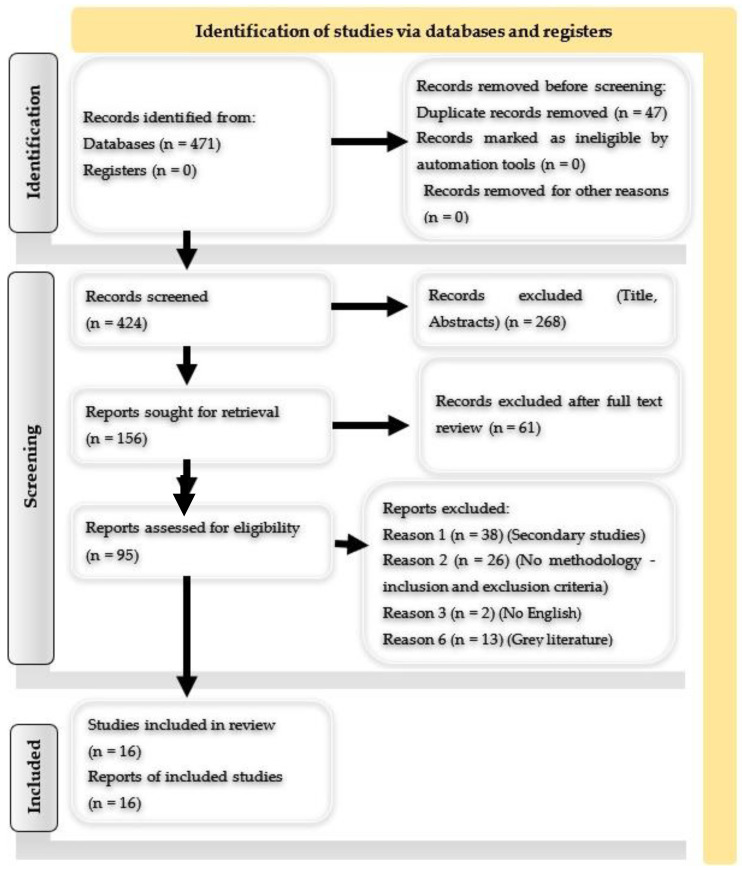
PRISMA flowchart.

From 471, 47 duplicates were removed. Thereafter, titles and abstracts of the remaining 424 articles were evaluated by three individuals for both relevance to the topic and research questions, of which 268 studies were excluded. Out of the 156 studies retrieved, 61 were unrelated to the study’s purpose and defined research questions. The review finally included 16 studies out of 95, due to the exclusion of the remaining 79 studies (n = 38 were excluded for inclusion criterion 1—were not secondary studies, n = 26 for inclusion criterion 2—did not have an appropriate methodology, n = 2 for inclusion criterion 3—were not in English, n = 13—included for exclusion criterion 6—gray literature). The process is summarized in Figure 1.

### 2.5. Data Collection and Extraction

The articles were collected, analyzed, and examined in detail using the Mendeley version 1.19.8 citation software. The research questions were answered by sorting the data through an Excel export form that included their main characteristics.

### 2.6. Morality

Due to conducting a secondary study, permission was not necessary. The views and findings of the studies included were analyzed objectively within ethical and moral guidelines.

## 3. Results

### 3.1. Descriptive Characteristics

Descriptive characteristics of the studies in this review, according to Table 4, included 16 studies that met the inclusion criteria from various academic databases. Specifically, the systematic review included 16 studies (Table 3), of which seven (7) were scoping review studies [1,11,58,88,89,90,91], four (4) were systematic reviews [85,92,93,94], three (3) were literature reviews [10,83,95], one (1) was a narrative review [84], and one (1) was a rapid review [96]. The geographic focus of the studies varied, with eight (8) of the studies reporting on a global scale [1,11,58,84,85,92,93,96], two (2) on the African continent [10,88], one (1) study related mainly to the USA and Australia, one (1) to Sub-Saharan Africa [90], one (1) to Southern Africa [94], one (1) to Russia [89], one (1) to the Caribbean [91], and one (1) to Charleston, South Carolina [95]. In terms of year of publication, four (4) studies were published by October 2024 [58,90,92,96], four (4) in 2022 [83,85,88,89], two in 2019 [10,94], two (2) in 2020 [84,91], two (2) in 2023 [11,93], one (1) in 2018 [95], and one (1) in 2021 [1].

### 3.2. Strategies and Practices That Enhance the Preparedness and Resilience of Health Systems

According to the studies included and captured in Table 5 of the systematic review, strategies to strengthen health system preparedness and resilience include actions to adapt to climate change in fifteen (15) studies [1,10,11,58,83,84,85,88,89,90,91,92,94,95,96], actions to mitigate climate change in one study [93], and six (6) studies related to both domains [1,11,84,89,90,93]. Ten studies refer more generally to addressing climate change and building resilient health systems [11,58,84,85,88,90,91,92,93,96], six (6) studies focus on preparedness for extreme weather events (drought, heavy rainfall) [1,10,89,91,94,95], and three (3) studies address mental health impacts and health systems’ coping strategies [1,84,91]. Also, three (3) studies focus on the impact on the healthcare workforce and its response [58,92,96], and one (1) study addresses the preparedness of emergency departments in fires [83].

To reduce healthcare emissions, it is important to promote policies and guidelines for interventions related to health threats [84,89]. The key focus of these policies should be on implementing climate change risk communication interventions and promoting environmental sustainability and psychological development and resilience [84,90]. Prevention and health promotion (via vaccination and improved hygiene) [1] will result in a decrease in the demand for health services [84,90]. In addition, environmentally friendly designs for heating, cooling, water, ventilation, and electrical services play an important role [11]. Finally, health care should concentrate on low-carbon activities, like using recyclable materials, properly distributing non-recyclable materials, promoting reusable products, enhancing air quality, and designing energy-efficient surgeries [93].

According to Skiner et al. (2022)’s study of adaptation to extreme climate change, to prevent future fire incidents in healthcare facilities, it is recommended to define major incident and mass casualty management plans, as well as address communication and workforce competency issues [83].

Studies suggest enhancing the resilience of individuals as a strategy for dealing with mental disorders [84]. Effective communication between patients and health professionals and patient self-management is the primary focus [91]. Moreover, community screening programs for mental disorders are implemented following extreme weather events [1,84]. The focus is on creating education and training programs for the workforce [84], collaborating with local institutions and pharmacists to establish drug distribution centers, and establishing a monitoring system for the medically vulnerable population [91].

Climate change affects the health system, its components, and its workforce [96]. Adaptable strategies include supporting the mental health of staff [58,92,96], providing education and training [10,58,92], fostering interdisciplinary cooperation, flexible roles, motivation [92], and enhancing transformational leadership [96].

**Figure 2:**
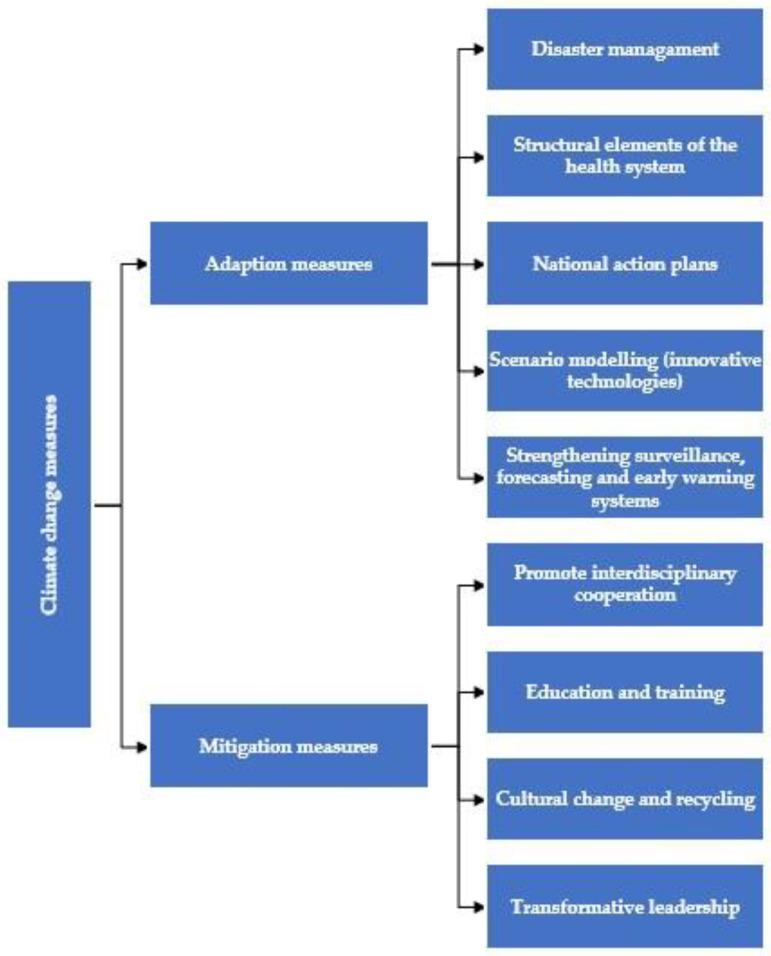
Climate change measures.

In terms of health system adaptation in general, strategies and resilience building relate to the building blocks of health systems, governance and leadership, service delivery, workforce, health information systems, and key medical products and technologies [11,96]. The most commonly cited strategies and interventions include developing a national action plan, formulating plans for heating, cooling, water, ventilation, and electricity services [11], and assessing the strengths and vulnerabilities of health services. Other actions involve strengthening disease surveillance systems [11,95], scenario modeling (expected to reduce the impacts that accompany extreme weather events) [95], information, capacity building, and early warning and forecasting systems for extreme weather events [90]. Moreover, the importance of awareness raising, political will, improved public health technologies and infrastructure, surveillance and research [10,89], and learning from past disasters is significant [85]. In this line, climate change provides an opportunity for health systems to implement strategies to build resilience against the impacts of environmental change [88] (Figure 2).

### 3.3. Key Barriers and Challenges to Strengthening the Preparedness, Sustainability, and Resilience of Healthcare Systems

Ten studies have emerged from the literature, as reported in Table 6 on the impacts, barriers, and challenges to the preparedness, sustainability, and resilience of health systems [10,58,83,85,88,91,92,93,94,95]. Five (5) studies refer to the impact of the health system [83,88,91,92,95], five (5) to health system barriers and challenges [58,85,92,94,95], five (5) studies to health impacts [10,83,88,91,95], and one (1) study on the carbon footprint of surgical interventions [93].

Climate change is causing serious health impacts through the spread of disease [10,95], increasing non-communicable diseases [10,83,91,95], injuries [91,95], drowning [95], burns, food insecurity [91], mental health disorders, and ultimately morbidity [91,95] and mortality [10,91]. These conditions contribute to an increase in the use and demand for health services [88,95] and the volume and severity of cases in emergency departments [83]. In addition, changing environmental conditions negatively affect the supply chain, quality, and health service delivery of health systems (through disruption of health care continuity, reduced access to health professionals and health care facilities [91], hospital evacuations, and power outages [92,95].

Furthermore, Word et al. (2024) and Zurinsky et al. (2024) report that there is a deficiency in preparedness planning and guidelines for natural disasters [58,92]. The disruption in service delivery occurs both during planning and response and recovery from catastrophic events [58]. The main problems are related to ambiguity in roles [58,92], coordination, communication, and information of workers [58]. Also observed are effects on occupational safety [92], the psychological and physical well-being of employees, and absenteeism (sickness, inability to access healthcare facilities) [58,92]. There is a lack of experience in nurse leadership in extreme situations and inadequate training for disaster preparedness [58]. Lack of awareness of environmental change among hospital administrators and decision-makers stems from gaps in understanding, preparation of healthcare organizations [95], and learning from previous extreme disasters [85]. Public health professionals lack an understanding of the epidemiological tools available to aid the local community in preparation. Additionally, a research gap was detected in crucial inquiries during catastrophic weather events and recovery due to the absence of funding and research protocols [95]. The review by Chersich et al. (2019) also includes all the aforementioned points. According to this, the health science curriculum in South Africa has been underdeveloped, and minimal adaptation measures have been implemented [94].

Lastly, healthcare facilities and activities are responsible for contributing to climate change due to CO_2_ emissions. The high rate of waste and energy consumption is related to surgical procedures in 2023, as suggested by Tsagkaris et al. (2023) [93]. This study is supported by the fact that the US financial sector produces an average of 126,030 kg of CO_2_ emissions for every $1 million invested [8]. In addition, in the Netherlands, the health system is responsible for 13% of raw material consumption, 4% of waste, and 7% of the total CO_2_ footprint [97].

Some of the barriers mentioned above were also found in the study by Opoku et al. (2021) [2] conducted in six African countries (Nigeria, Ghana, South Africa, Ethiopia, Kenya, and Namibia). Specifically, the study found the failure of governments to partner with organizations, to ensure adequate human resource training in health systems to address climate change. In addition, it confirmed limited funding, reduced workforce, and limited hospital and primary health care facilities. Finally, reduced availability of technical and material resources and ineffective surveillance systems were observed.

### 3.4. Visualization of Results

VOSviewer version 1.6.20 software was used to develop a network of keywords, retrieved from relevant articles on the health impacts of climate change and strategies for adaptation and mitigation, and the barriers to their implementation. The resulting bibliometric networks are weighed and indicate that the lines represent both the strength of the relationship (by thickness) of the nodes and their connectivity. This software was chosen because it is user-friendly and has no charge, and the results of its output are valid. More specifically, it ensures repeatability since users can access data [98]. In addition, it is a tool that, due to its node size, distance, and time-based approach, has characteristics that meet the visualization requirements of bibliometric networks. This finding also follows from the fact that visualized maps in the form of clusters are easy to both analyze and interpret. More specifically, a keyword that has a larger circle has more links, and the thickness of the line indicates the connection between the keywords. Namely, the greater the thickness, the stronger the connection between the keywords [99].

**Figure 3:**
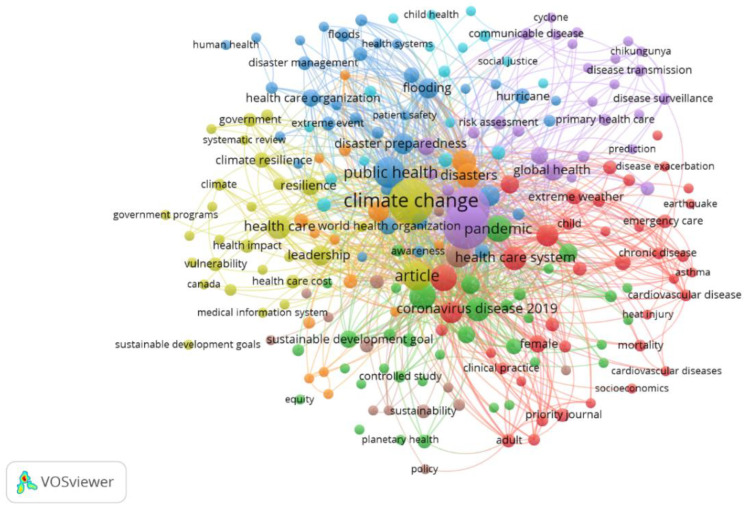
Co-occurrence network map of keywords.

**Figure 4:**
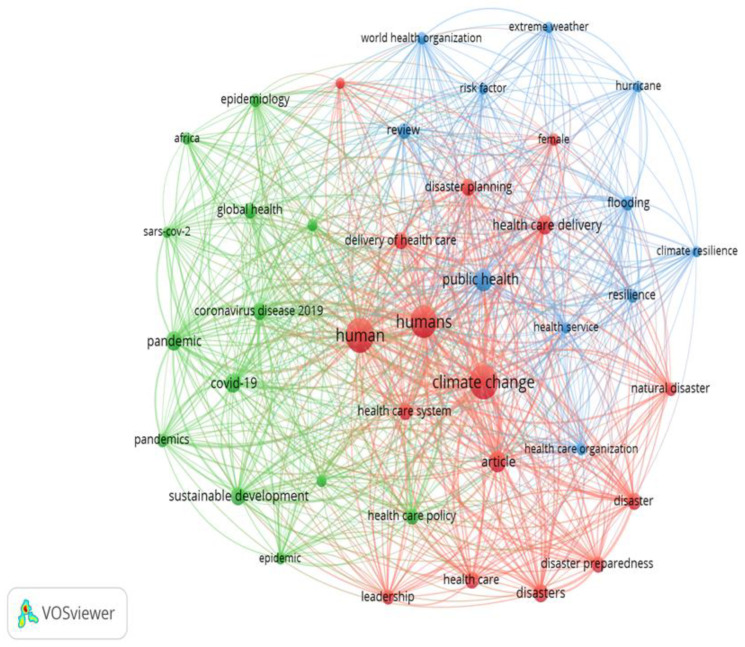
Network map of keyword co-occurrence with a minimum of 5 keywords. Coexistence net map with at least 5 keywords.

The keyword co-occurrence network map shows the 852 most frequently used keywords (Figure 3). The network clusters in the map are shown in different colors, indicating that data clustered in the clusters with the above characteristics are identified with a higher level of association. To select, preserve, and visualize the most relevant concepts, a bibliometric analysis was also performed, where the number 5 was set as the minimum occurrence of keywords (Figure 4). In total, 40 keywords met the lower limit in the analysis out of a total of 852. The map therefore shows that clusters illustrate the strong correlation between climate change, healthcare, and sustainability.

## 4. Discussion

This systematic review, using a methodologically consistent approach, attempted to map, assess, and synthesize practices and strategies that healthcare systems should follow to address the challenges posed by climate change. The implementation of these preparedness strategies encounters obstacles due to multiple factors, such as the intensity of events and the rise in both non-communicable and communicable diseases and injuries. These factors, combined with the increase in demand for services, lack of operational planning, and strengthening of the structural components of health systems (financing, governance and leadership, service delivery, workforce, health information systems, and essential medical products and technologies) are putting their sustainability at risk.

Specifically, to build climate resilience in the health system, it is proposed to create a climate capital lens that includes risk assessment and synergies among stakeholders such as public health, academia–research, and the Centers for Disease Control and Prevention [100,101]. More precisely, increased funding should be allocated to build resilient infrastructure [4,100,101], and provide services [101,102]. Additionally, appropriate planning should be conducted based on population needs [100,103], and health professionals’ knowledge and capacity should be built [23,100,101,102]. Therefore, according to Abbas et al. 2022, climate change is a complex challenge at the global level, affecting the whole range of ecological, socio-political, environmental, and socio-economic sectors [104].

A study by Rawat et al. (2022) on the 2015–2016 drought in Ethiopia showed that improving health system resilience requires government leadership to strengthen decentralized decision-making [23]. In addition, effective preparedness requires proper planning [61], identification, and support of vulnerable populations [102]. It also requires the development of response plans [4,61,102], epidemiological and entomological surveillance, and epidemic early warning systems [105]. Health systems require an increase in financial resources with a reasonable distribution [61,102] and reinforcement with accurate data drawn from evidence beyond previous experience. Governments, utilities, insurance companies, environmental bodies, and universities can provide accurate data. Similarly, external experts should carry out the evaluation, selection, and interpretation of scenarios [61]. According to Pley et al. (2021), technological and digital innovations are contributing to the integration of climate data [106], enabling health surveillance and information systems to detect communicable diseases [101,106] and weather conditions [101,102]. In addition, AI reduces the waste of scarce healthcare resources and improves the quality and efficiency of services (less time spent on diagnosing and treating patients). At the same time, it facilitates the adoption of sustainable practices, such as telemedicine [96,107].

In terms of mitigation, healthcare facilities should aim to reduce carbon emissions [7,102]. The pursuit of this is linked to waste reduction [4], energy use, and the supply chain [102]. Additionally, improving population health by preventing, promoting, and managing chronic diseases reduces the need for health services and resources (human and material) [61]. In conclusion, it is important to avoid providing care that is not beneficial to the patient [66,97] and to consider the environmental impact of various healthcare intervention options [97]. All of the above findings, related to mitigation and adaptation, are consistent with the WHO’s Framework for Climate Resilient Health Systems, which specifically states that resilient and environmentally sustainable facilities are an important component of universal health coverage [108]. The implementation of adaptation and mitigation strategies necessitates a sufficient number of skilled human resources, adequate working conditions [109], and awareness of sustainable practices and climate change in general [13,110].

The National Health Service (NHS) in the UK leads the way in terms of the policies that health systems follow for sustainability because it has a well-organized system of governance and accountability, allocates resources, develops appropriate strategies, passes appropriate legislation, and continuously evaluates its systems [111]. This strategy resulted in a 21% decrease in water use between 2010 and 2017 and an 18.5% decrease in carbon emissions during the same period while committing to achieving zero carbon emissions by 2040 [112].

Nhamo & Muchuru (2019) conducted an investigation into the implementation of adaptation measures in 18 English-speaking countries on the African continent. Their findings showed that there were systems in place for forecasting and early warning of extreme weather events. In addition, infectious disease surveillance systems are functioning, research is being strengthened, public health infrastructure and technology are being enhanced, adaptation policies are being implemented, and the public is being educated and sensitized to environmental issues [10]. However, health systems across the African continent are under immense pressure due to their socioeconomic conditions [2]. The implementation of the Paris Agreement led Iran to adopt the above adaptation measures and policies [113].

A study by Älgå et al. (2018) on the preparedness of primary healthcare facilities in Vietnam found that 90% of facilities had flood protection plans, 40% received flood preparedness training, and 60% reported inadequate training and support for preparedness. The funding for 50% of structures was raised to prepare for flood contingency [114]. According to Austin et al. (2016), OECD governments are focusing on the risks related to infectious diseases and heat-related risks arising from climate change [115].

Climate change’s direct or indirect public health problems create barriers to tackling it [4]. In particular, the immediate issues arise from events including heatwaves [4,101,103,116], storms, hurricanes [4], increased rainfall [101], floods [61], fires [4], and droughts [4,61,116]. Similarly, indirect situations are created by changes in water quality, air quality, and threatened food safety supply [101]. These situations lead to excessive morbidity and mortality from both communicable and non-communicable diseases and an increase in the number of people living with disabilities, which contributes to the loss of productivity and an increase in direct and indirect costs (social and health care) [4].

It is estimated that climate change has caused the death of over 150,000 people and caused 5.5 million disability-adjusted life years annually since 1970 [10]. This excess mortality is expected to increase by 2050, and heat exposure is expected to lead to more than 100,000 deaths per year [117]. Research by Sarfaty et al. (2016) found that both international and US physicians observed climate change impacts on their patients’ health. Specifically, international physicians were more likely to report experiencing health events related to heat (69% vs. 48% of US physicians), vector-borne infections such as mosquitoes (59% vs. 49%), weather-related injuries (69% vs. 57%), and diarrhea due to foodborne or waterborne illness following rainfall or flooding (55% vs. 26%). [118]. In terms of mental health, research by Matthews et al. (2019) found that post-traumatic stress was more pronounced in members of the population whose home, business, or farm was flooded. Socio-economically marginalized people were more likely to have their homes flooded and displaced [119].

On this basis, risks and pressures are constantly increasing for health systems and healthcare facilities, leading to a reduction in the capacity of the health workforce to carry out their task of protecting people [109]. In particular, crises created by environmental change increase the demand for health services [61,120], which affects their ability to continue their activities during these events [61,120,121] and compromises their financial viability [100], access, and quality of health services [100,120,122]. Extreme weather events can lead to potential issues with access to healthcare facilities, facility damage, power and transportation outages, increased illness and injury rates, higher rates of illness and injury [9,61,120], and disruptions to the supply chain [9,61,120,122]. During the acute phase of an extreme weather event, the number of deaths and services used in emergency departments increase indirectly due to physical injuries, drownings, and electrocution. The growing number of affected populations resulted in the need for more staff [9,120] to deal with physical and mental disorders and longer waiting times [9]. Due to the aforementioned situation, there is a rise in medical mistakes and patient vulnerability, as well as delays in cancer detection and management [122].

However, all of these obstacles and challenges are due to a lack of preparedness, coordination, planning, training, inadequate resource mobilization, failure to provide early warning to vulnerable populations [123], and, most importantly, negligible and inadequate funding [101,124]. On this basis and based on what has been mentioned, the lack of financial resources persists globally, despite increased awareness. This may be possibly due to the industrial capitalist lifestyle of Western populations. In many cases, the public sector does not have the expertise to implement policies, actions and activities, that lead to economic growth and the reduction of social inequalities. For this reason, it is suggested that the private sector should fill the gap left by the public sector through partnerships.

Research has shown that the health system is unable to effectively deal with climate change due to a lack of resources [65,125,126,127,128], staff training [125], adequate information [126], leadership [127], coordination [65], and preparedness [2,65], as well as poor planning [65] and limited public and health professional awareness [127]. The research findings above are supported by a study by Baumann et al. [5]. Anesthetists reported a lack of knowledge about issues related to the carbon footprint of drugs and medical products and had limited time to think about their environmental footprint during their activities [5].

On 14 March 2019, Beira, the largest city in Mozambique’s Sofala province, was affected by Cyclone Idai, which confirms this. Health services were disrupted due to the destruction of road infrastructure and health facilities. Moreover, malnutrition was caused by the devastation of agricultural production and an increase in the incidence of malaria and cholera epidemics [121]. In the same context, following flooding in Boston, Lincolnshire, all health services were disrupted, putting great pressure on the system and reducing its ability to respond to climate extremes and provide routine healthcare [120]. Health facilities were severely damaged by the Alberta floods (2013), Hurricane Sandy (2013), Hurricane Juan in Nova Scotia (2003), Hurricane Katrina (2002), and the ice storm in eastern Canada (1998). Access difficulties, increased workload, supply chain problems (medicines), power outages, and injuries have emerged [129].

It is clear from the above that climate change disproportionately affects vulnerable populations, such as people with chronic diseases, the elderly, the poor and people with disabilities [101,130]. This impact is particularly pronounced in countries with inadequate or less developed health systems, as the climate crisis exacerbates existing social inequalities and widens the gap [131]. Vulnerable people are at greater risk due to their reduced capacity to act, and their increased vulnerability [101]. For instance, people of color and poor people in the U.S. are at greater risk to their health, while their contribution to greenhouse gas emissions is minimal [100]. It has also been reported in the literature that the most vulnerable populations at risk are rural populations, especially women in developing countries, where their livelihoods are highly dependent on the resources they produce and the weather [132].

Additionally, the vulnerability of the Global South consists of two factors: exposure to hazards and inadequate resources to protect against them [133]. This finding also follows from the fact that the African continent is particularly vulnerable to extreme weather events, due to weak adaptive capacity, lack of early warning systems, an already overburdened health system, lack of and inadequate infrastructure, and above all, poverty [88]. It is worth noting that areas in the Horn of Africa (Ethiopia, Somalia, Kenya) are particularly vulnerable to the effects of climate change (prolonged drought, higher temperature, heavy rainfall) forcing people to leave their homes and become internally displaced or cross-border refugees of residence [63]. This report and the previous sections show that further delays in implementing the necessary commitments by countries to address climate change, in line with the objectives of the Paris Agreement, are increasingly damaging people’s health. [134]. For climate justice to exist, it is suggested that policies for both mitigation and adaptation should be developed and implemented [133]. First and foremost, political will is required and putting health at the center through cross-sectoral collaboration in all climate change policies at international, national, and local levels [134]. In addition, it requires alignment with the demands of society as a whole, creating joint planning, engaging stakeholders at the local level, and continuous monitoring and updating of policies and guidelines, that include climate change impacts [135]. Moreover, investment in clean energy and renewable energy sources could provide power in developing countries and remote areas [134]. Finally, building resilient health systems requires investment in the building blocks of the health system [17] and the adoption of the World Health Organization’s guidelines for resilient and environmentally sustainable healthcare facilities [108]. In terms of implementing policies and interventions, it is useful to document some good practices. The Centers for Disease Control and Prevention (CDC) has developed a framework called Building Resilience Against Climate Effects, or BRACE. This tool supports public health adaptation efforts at the regional and local levels and helps assess vulnerability, select interventions, and model the potential health impacts of climate change [136]. The Lancet Countdown has developed indicators (adaptation, mitigation) to monitor progress in the health sector [137]. At the European level, the European Commission announced in 2021 the strategy to be followed by EU Member States through the Green Deal [138]. Through this and the European Climate and Health Observatory, information will be provided to Member States and information exchange, and cooperation will be promoted. Member States, will be required to report to the Commission every two years on their adaptation strategies, national planning, and a description of measures implemented and planned [139].

Finally, it is important that all mitigation and adaptation measures be accompanied by an economic assessment (cost–benefit). This will help developing countries make rational decisions for a more efficient allocation of resources [140].

## 5. Conclusions

Climate change, despite having existed for many years, is today a global challenge, as mentioned earlier. The lack of interest from the scientific community has highlighted the need to deepen scientific research, at the level of primary and secondary studies. It is recommended that longitudinal studies on the resilience of health systems be adopted and integrated at the scientific level, emphasizing a multidisciplinary approach to assess and predict future health impacts. This research should focus on identifying the benefits of strategies, related to adaptation, mitigation, preparedness, and resilience. The gap between policy and intervention can be mitigated by making adaptive and transformative changes, needed to effectively manage healthcare systems in times of crisis.

### 5.1. Suggestions

This paper focused on reviews related to the impacts of climate change on health and health systems. It is suggested that future studies should focus on empirical research both on the topic addressed in the paper and on the views of health professionals and senior health facility managers (using mixed methods—quantitative and qualitative analysis), to examine impacts and solutions in the immediate and longer term. These studies will contribute to a deeper understanding of the impacts of climate change and interdisciplinary collaboration (economists, health professionals, sociologists) for the implementation, evaluation, and effectiveness of policies and protocols for adaptation and mitigation. It is also proposed to implement educational programs, incorporating innovative technologies to raise awareness of environmental sustainability issues among the community and health sciences. Developing methods to measure the sustainability and resilience of the health system using modern technology (artificial intelligence) is also essential. In addition, it is proposed to fully develop and invest in health care facilities in modern technologies, using artificial intelligence for health education, prevention, treatment, and rehabilitation of diseases (radiological diagnostics, telemedicine).

It is recommended that the perspectives of health professionals, patients, and the community be incorporated into future studies to ensure the feasibility of proposed solutions and to deepen research in underrepresented countries, regions, and vulnerable populations. Longitudinal studies should also be conducted to help monitor the long-term effectiveness of policies as well as long-term health impacts, particularly in areas prone to climate-related disasters (small island states). Policymakers need to work with the community and their intervention strategy should respond to the requirements of the community at national, regional, and local levels. It is proposed to further invest in renewable energy and infrastructure, thereby enhancing the resilience and sustainability of the health system. Finally, to manage the financial resources of interventions more efficiently, it is recommended that economic evaluations (cost-benefit, cost-effectiveness) be carried out.

### 5.2. Limitations and Areas for Future Research

This study acknowledges the limitations in the scope of the studies included and the criteria used to select them. Therefore, to ensure the reliability and validity of the study, it is necessary to acknowledge its limitations. The inclusion and exclusion criteria used in the study should be more clearly stated. In addition, any potential bias or limitations in the scope of the included studies should be identified. The article by Mosadeghrad et al. (2023) presents a chart of the studies without explaining the reasons for their inclusion or exclusion. The literature search was conducted in both English and Persian, which may have limited the inclusion of studies implemented in other languages. An Iranian database was searched, which does not have an English translation, potentially raising issues of bias by the authors [11]. Grigorieva & Livenets in their study did not impose any restrictions and did not present a flowchart. However, they reported that they removed duplicate articles and excluded studies based on title, abstract, and results [89].

The limitations of this study were addressed by including studies with methodological problems because they followed some parts of a guideline reference. In particular, if they reported inclusion and exclusion criteria and did not have a flowchart, or if there were incomplete references in the flowchart, this could raise concerns about bias. In addition, the requirement to include only studies published in English with peer review could limit valuable information about the overall picture that could be gathered to answer research questions. A search strategy in the widely used databases (Google Scholar, Scopus, PubMed) may limit the articles that could be included in the study.

The exclusion of gray literature may have potentially affected the completeness of the results because important references from organizations and from unpublished studies may have been excluded. Similarly, the time limitation of the inclusion and exclusion criteria may not have taken into account studies that are still relevant. These limitations mentioned may affect the results of the review in terms of the overall picture and generalizability of the findings. It is suggested that future studies should expand the databases, include gray literature, and not be limited to English language studies.

The majority of the studies in the paper are theoretical, with a focus on the preparedness of health service organizations and health systems in general. In addition, most of the studies in the paper are largely theoretical in nature regarding the preparedness of health service organizations (and health systems in general) to cope with climate change. Consequently, the proposed adaptation and mitigation measures are not evaluated in real-life settings and a gap for further research is created.

In particular, it is recommended that longitudinal studies be carried out to help monitor the long-term effectiveness of policies and possible policy modifications. Finally, it is proposed to conduct longitudinal studies to monitor the long-term impacts of interventions, especially in regions prone to climate-related disasters (small island states).

## Figures and Tables

**Table 1:** Search strategy.

Database	Search Strategy	Date of Search	Year of Issue
Google Scholar	Use of the following combination of relevant keywords: (“Climate Change” or “Environmental change”), (“Health Systems or Health care”), (“Readiness or Preparedness”), (“Sustainable or Resilient”).	21 April, 15 June, 9 September 2024	2018–2024
PubMed	Use of the following combination of relevant keywords: (“Climate Change” or “Environmental change”), (“Health Systems or Health care”), (“Readiness or Preparedness”), (“Sustainable or Resilient”).		
Scopus	Use of the following combination of related keywords: “Climate Change” or “Environmental change”, “Health Systems” or “Health care”, “Readiness” or “Preparedness”, “Sustainable” or “Resilient”.		

**Table 2:** Exclusion criteria and their justification.

Description	Justification
Secondary studies	Comprehensively reflect the existing literature
Studies using inclusion and exclusion criteria in their methodology	Give greater validity and credibility
Studies published in English	Dominant worldwide in the scientific community
Published between 2018–2024	The defined time frame helps to ensure repeatability and to identify the most up-to-date literature
Unrelated content	To enable their evaluation
Books and gray literature (theses, conference proceedings, reports)	Difficulty in evaluating them

**Table 3:** Number of articles in the databases.

Database	References
Google Scholar	301
PubMed	113
Scopus	57

**Table 4:** Description of systematic review studies.

A/A	Authors	Year	Title	Type of Study	Citations (n)	Important Points	Reporting Location
1.	Skinner et al. [83]	2022	A Literature Review on the Impact of Wildfires on Emergency Departments: Enhancing Disaster Preparedness	Literature Review	15	(1) Fires: serious health impacts on the population, increase in the volume/severity of emergency room cases. (2) Recommendations/findings: communication, human resource adequacy, definition of major incident/massive disaster management plans.	USA, Australia
2.	Palinkas et al. [84]	2020	Strategies for Delivering Mental Health Services in Response to Global Climate Change: A Narrative Review	Narrative Review	13	(1) Climate change affects the mental health of the population. (2) Coping strategies: (a) strengthening individual and community resilience through programs to monitor mental disorders; (b) creating education and training programs for the workforce; (c) adopting mitigation policies and guidelines for interventions related to threats to health and well-being (population displacement, civil conflict); (d) implementing interventions to communicate the risk of climate change	General
3.	Theron et al. [88]	2022	Climate change and emergency care in Africa: A scoping review	Scope Review	17	(1) Health impacts contribute to increased use of health services and resources. (2) Create opportunities to engage health systems to improve emergency preparedness.	Africa
4.	Borg et al. [1]	2021	Climate change and health in urban informal settlements in low- and middle-income countries—a scoping review of health impacts and adaptation strategies	Scope Review	27	(1) Climate change (extreme heat, flooding) exposes the population of informal urban settlements to health risks. (2) Access to mental health services could be facilitated through screening for depression and domestic violence following extreme weather events. (3) Adaptation interventions: (a) immunization through vaccination, (b) improved hygiene, (c) community awareness of risks (e.g., heatwaves), and (d) capacity building of the health workforce.	General
5.	Ward, A. et al. [58]	2024	Enhancing primary healthcare nurses’ preparedness for climate-induced extreme weather events	Scope Review	0	(1) Climate change requires primary care nurses to be prepared for extreme weather events. (2) Barriers and challenges: (a) lack of experience of nurse leadership; (b) disruption of health service delivery; (c) lack of information, plans, and guidelines; (d) lack of staffing, communication, and coordination among health professionals and disagreements over role allocation; (e) inadequate training for disaster preparedness; (f) burden on physical and mental health and inability to access infrastructure and medical supplies. (3) Response: (a) mental and physical health support for nurses; (b) detailed planning; (c) education and training; (d) funding.	General
6.	Ali et al. [85]	2022	Investigating Organizational Learning and Adaptations for Improved Disaster Response Towards “Resilient Hospitals:” An Integrative Literature Review	Systematic Review	8	(1) Nine (9) areas of learning for hospitals from previous disasters were identified: (a) leadership/governance, (b) planning and risk assessment (c) supervision and monitoring, (d) effective communication and engagement, (e) workforce safety (f) appropriate equipment and resources, (g) infrastructure and facilities (h) implementation of innovation and learning and (i) evaluation. (2) In implementing the Deming cycle, a few studies (4) described a comprehensive cycle of hospitals learning from previous disasters.	General
7.	Mosadeghra et al. [11]	2023	Strategies to strengthen a climate-resilient health system: a scoping review	Scope Review	25	(1) A total of 87 actions for building a climate-resilient health system were identified, classified into 6 thematic areas. (2) The domains are related to the building blocks of health systems (financing, governance and leadership, service delivery, workforce, health information systems, and key medical products and technologies). (3) The most frequent actions include (a) developing a national action plan for climate change adaptation (b) formulating plans for heating, cooling, water, ventilation, and electricity services, (c) assessing the capacities and vulnerabilities of health services, and (d) strengthening disease surveillance systems.	General
8.	Zurynski et al. [92]	2024	Bolstering health systems to cope with the impacts of climate change events: A review of the evidence on workforce planning, upskilling, and capacity building	Systematic Review	1	(1) The impacts of climate change on the health workforce include (a) system collapse, (b) job security, (c) psychological well-being, and (d) absenteeism due to access problems and illness). (2) Responses to challenges: (a) staff education and training, (b) interdisciplinary collaboration, (c) role flexibility, (d) motivation, (e) psychological support, and (f) appropriate planning for the ability to adapt to extreme situations	General
9.	Grigorieva & Livenets [89]	2022	Risks to the Health of Russian Population from Floods and Droughts in 2010–2020: A Scoping Review.	Scope Review	3	(1) Russia is frequently affected by extreme climatic and weather events, which affects both directly and indirectly the health and well-being of the population. (2) Some health impacts can be avoided: (a) through the development of early warning systems, (b) through the preparedness, resilience and response of public health and health systems to climate change.	Russia
10.	Runkle et al. [95]	2018	Population health adaptation approaches to the increasing severity and frequency of weather-related disasters resulting from our changing climate: a literature review and application to Charleston, South Carolina	Literature Review	38	(1) Heavy rainfall and hurricanes affect the health and well-being of the population and health systems (evacuations of hospitals and areas, power outages, drowning, injuries, mental health disorders, stress, gastrointestinal and vector-borne diseases, exacerbation of chronic diseases, low birth weight and premature births, reduction in quality and possible disruption of health services, and increase in demand for health services) (2) Public health vulnerability results largely from the following: (a) lack of awareness of environmental change issues among hospital administrators and decision makers; (b) lack of understanding by public health professionals of the epidemiological tools available to assist local communities in preparation; (c) inability to answer important questions during catastrophic weather events and during recovery due to unavailability of funding and research. (3) Addressing these situations and appropriate planning for vulnerability, disease surveillance and scenario modelling will reduce the impacts that accompany extreme weather events.	Charleston, South Carolina
11.	Tsakonas et al. [96]	2024	Rapid review of the impacts of climate change on the health system workforce and implications for action	Rapid Review	0	(1) Impacts: climate change affects the health system, its components, and therefore its workforce. (2) Adaptive coping strategies include (a) psychosocial support for the workforce, (b) strengthening transformational leadership, (c) planning and emergency preparedness of their structural components (leadership, workforce, information systems, key medical products and technologies, financing).	General
12.	Tsagkaris et al. [93]	2023	Eco-Friendly and COVID-19 Friendly? Decreasing the Carbon Footprint of the Operating Room in the COVID-19 Era	Systematic Review	1	(1) Surgeries are associated with a high rate of waste and energy use. (2) Remedy: (a) improvement in air quality, (b) use of recyclable materials and proper distribution of non-recyclables, and (c) energy-efficient surgical design.	General
13.	Hounkpatin et al. [90]	2024	How are health systems in Sub-Saharan Africa adapting to protect human health from climate change threats? A scoping review and case study	Scope review and case study	1	Adaptation measures are identified in seven policy areas: (a) health systems strengthening, (b) financing, (c) information and capacity building, (d) programming, (e) social resilience development policies, (f) mitigation and prevention, and (g) disaster preparedness, response, and recovery.	Sub-Saharan Africa
14.	Nhamo & Muchuru [10]	2019	Climate adaptation in the public health sector in Africa: Evidence from United Nations Framework Convention on Climate Change National Communications	Literature review and grounded theory	34	(1) Environmental change favors the spread of diseases such as cholera, typhoid, and malaria, and extreme heat and cold waves increase mortality. (2) Key adaptation measures relate to the following: (a) early warning and forecasting systems for extreme weather events, (b) awareness raising and education, (c) political will, (d) improving public health technologies and infrastructure, (e) surveillance and strengthening research.	Africa
15.	Chersich & Wright [94]	2019	Climate change adaptation in South Africa: a case study on the role of the health sector	Systematic Review	125	(1) There are well-defined systems for monitoring extreme weather events and infection surveillance. (2) Barriers and challenges: (a) there are no accurate data to assess the preparedness and response of the already burdened health system to climate extremes, (b) there is not adequate attention in health science curricula, and (c) the adaptation measures adopted are minimal.	South Africa
16.	Hassan et al. [91]	2020	Management Of Chronic Noncommunicable Diseases After Natural Disasters in the Caribbean: A Scoping Review	Review Article	8	(1) Impact: natural disasters increase morbidity and mortality from chronic non-communicable diseases (2) Challenges: disruption of continuity of health care, food insecurity and exacerbation of chronic diseases (3) Response strategies: (a) working with local institutions and pharmacists to establish drug distribution centers, (b) effective patient–health professional communication, (c) empowering self-management of illnesses (physical and mental) both before and after disasters, (d) establishing a monitoring system for medically vulnerable people.	The Caribbean

**Table 5:** Strategies to strengthen health system preparedness and resilience.

Actions, Strategies	Studies—References
Strategies for adapting to climate change	Borg et al. (2021) [1], Nhamo et al. (2019) [10], Mosadeghrad et al. (2023) [11], Ward et al. (2024) [58], Theron et al. (2022) [88], Grigorieva et al. (2022) [89], Hounkpatin et al. (2024) [90], Hassan et al. (2020) [91], Ali et al. (2022) [85], Zurynski et al. (2024) [92], Chersich et al. (2019) [94], Skinner et al. (2022) [83], Runkle et al. (2018) [95], Palinkas et al. (2020) [84], Tsakonas et al. (2024) [96]
Strategies for climate change mitigation	Tsagkaris et al. (2023) [93]
Studies related to both mitigation and adaptation to climate change	Borg et al. (2021) [1], Mosadeghrad et al. (2023) [11], Grigorieva et al. (2022) [89], Hounkpatin et al. (2024) [90], Tsagkaris et al. (2023) [93], Palinkas et al. (2020) [84]

**Table 6:** Studies on the impacts, barriers, and challenges to the preparedness, sustainability, and resilience of healthcare systems.

Authors	Studies
Runkle et al. [95]	Impact on Health, Impact on the Health System, Barriers, Challenges to the Health System
Nhamo & Muchuru [10]	Effects on Health
Theron et al. [88]	Effects on Health and Effects on the Health System
Ward et al. [58]	Obstacles and Challenges in the Health System
Hassan et al. [91]	Effects on Health
Ali et al. [85]	Obstacles and Challenges in the Health System
Zurynski et al. [92]	Effects on the Health System, Barriers, Challenges to the Health System
Tsagkaris et al. [93]	Carbon Footprint from Surgical Procedures
Chersich & Wright [94]	Obstacles and Challenges in the Health System
Skiner et al. [83]	Effects on Health and Effects on the Health System

## Data Availability

Data are available upon request.
